# Sleep Well, Study Well: A Systematic Review of Longitudinal Studies on the Interplay between Sleep and School Experience in Adolescence

**DOI:** 10.3390/ijerph20064829

**Published:** 2023-03-09

**Authors:** Valeria Bacaro, Liesbeth Carpentier, Elisabetta Crocetti

**Affiliations:** 1 Department of Psychology “Renzo Canestrari”, Alma Mater Studiorum Università di Bologna, 47521 Cesena, Italy; 2 Faculty of Psychology and Educational Sciences, Katholieke Universiteit Leuven, 3000 Leuven, Belgium

**Keywords:** adolescence, sleep, school, systematic review, longitudinal

## Abstract

Adolescents spend most of their daily time in school and performing school-related activities. Different aspects of their school experiences, such as school performance, psychological factors related to school, and structural factors, consistently impact adolescents’ health and are likely to be intertwined with their sleep (i.e., quantity and quality, sleep disturbances). This systematic review aimed to comprehensively summarize the reciprocal and longitudinal associations between adolescents’ sleep and multiple aspects of their school experience. Using multiple search strategies and applying a two-step selection process, 25 journal articles matched the eligibility criteria and were thus included in the review. The results highlighted the contribution of poor sleep quality and sleep disturbances in predicting longitudinal school experiences-related outcomes (i.e., decreasing school engagement and performance, and increasing school-related burnout, absenteeism, and bullying). At the same time, the results showed how experiences related to the school’s psychological factors (e.g., high levels of school burnout and stressful environment) and structural characteristics (e.g., early school entrance time) affect youth sleep over time (i.e., decreasing sleep quality and quantity). These main findings provided novel insights into the bidirectional relationship between school experience and sleep health, highlighting the importance of more longitudinal research investigating all aspects of healthy sleep, including the size and direction of the association.

## 1. Introduction

Adolescence is a life period in which changes happen at multiple levels (i.e., physical, cognitive, and social). These modifications involve different developmental domains, such as identity formation, personal relationships with parents and peers, and internalizing and externalizing problems that can affect adolescents’ adjustment [1]. In this vein, it is worth considering that sleep is one of the central aspects of adolescents’ well-being [2]. Healthy sleep patterns are a combination of adequate sleep duration, good sleep quality, and regular sleep timing [3,4]. Nevertheless, according to the National Sleep Foundation, most of today's adolescents are sleep-deprived [5]. This condition of sleep deprivation and poor sleep quality in adolescence has been linked to several adverse health outcomes [6], and behavioral and emotional problems.

Given this centrality of healthy sleep patterns for adolescents’ well-being, it is of the utmost importance to understand, on the one hand, which factors can affect sleep and, on the other, how sleep patterns influence other aspects of their lives. In this regard, adolescents are embedded in different social contexts that could impact their sleep, and school is a key social factor [2]. Thus, their school experience is likely to be intertwined with their sleep patterns. In line with this reasoning, the current systematic review aims to comprehensively summarize the longitudinal research on the bidirectional relationships between sleep and school experience in adolescents.

### 1.1. Sleep in Adolescence

Healthy sleep is a multifaceted construct composed of several aspects, including quantitative (i.e., sleep quantity and timing) and qualitative (i.e., continuity and self-satisfaction with sleep) factors, along with sleep disturbances that combine both elements [4] (cf. Table 1). 

Regarding the quantitative aspects, sleep duration is defined as the number of hours slept at night. It is closely related to the sleep schedule, which refers to individuals’ bedtimes and wake-up times. During adolescence, sleep duration tends to decrease [7]. The Perfect Storm model of adolescent sleep [8] underlines an interplay between bioregulatory mechanisms and psychosocial factors that contribute to creating a deficient environment for adolescents’ sleep health (e.g., late-night screen time, newfound autonomy in choosing their bedtime, school start times, social engagement, and caffeine intake). For this reason, adolescents often may not experience the recommended amount of sleep (8 to 10 h following the National Sleep Foundation guidelines) [5]. This decline is particularly alarming because short sleep duration has been associated with multiple adverse outcomes, such as an increased risk of depressed mood and impaired cognitive functioning [9,10]. 

With regards to the qualitative aspect, the National Sleep Foundation identified several key indices of sleep quality: the sleep onset latency (the amount of time it takes to fall asleep), the number of awakenings, the wake after sleep onset, and sleep efficiency (defined as the time in bed that was spent sleeping). Poor sleep quality is a common problem in adolescence [11] and has short- and long-term adverse health outcomes, including risk for obesity, cognitive impairment, and mental health problems [12,13,14]. Furthermore, at the same time, typical conditions belonging to extrinsic factors, such as early school schedules and evening extracurricular activities, are also known to alter youths’ sleep quality [15].

Finally, it is essential to consider the presence of sleep disturbances during adolescence. Pediatric insomnia has been defined as a repeated difficulty with sleep initiation, duration, consolidation, or quality that occurs despite age-appropriate time and opportunity for sleep [16]. Sleep difficulties among youths are recognized as an international public health issue since they are bidirectionally associated with their physical and mental health [17,18,19]. Despite this sleep health impairment, these problems are often not adequately diagnosed and treated in pediatric populations [20]. 

A growing literature has highlighted the negative consequences of limited sleep duration, poor sleep quality, and sleep disturbances on overall mental and physical health in adolescence. At the same time, it is of the utmost importance to consider the diverse aspects of adolescents’ everyday life that could affect their health and sleep. In particular, since adolescents spend a significant amount of time in school, the school experience represents a complex social context that should be considered in evaluating sleep health. 

### 1.2. School Experience in Adolescence

School is one of the foremost socialization environments influencing adolescents’ development [21]. School experience is pervasive in adolescents’ lives, constituting the setting in which they spend most of their waking hours. Several aspects of the school experience can be developmentally related to adolescents’ sleep, such as school performance, psychological aspects linked to the school’s social context, and the school structure and environment characteristics. 

One of the school experience’s central angles is school performance (expressed through school grades). A meta-analysis of cross-sectional studies [22] showed that higher sleep duration, better sleep quality, and minimal daytime sleepiness were modestly but significantly related to better school performance (with more substantial effects related to sleep quality). Moreover, previous studies also showed how sleep could be crucial in maintaining learning efficiency [23]. However, this evidence needs to be corroborated with longitudinal studies that can disentangle the main direction of effects. Notably, it remains unclear how the potentially bidirectional association between sleep and school performance unfolds over time and which is the main direction of effects. 

Furthermore, it is of the utmost importance to consider the psychological aspects of the school experience that affect and, in turn, can be affected by sleep health patterns during adolescence. The psychological factors related to the school experience could be differentiated into negative and positive. Specifically, with regard to the negative aspects, adolescents could experience various school-related problems. For example, school burnout (a combination of exhaustion because of the school demands, cynicism, and feelings of incompetence as a student) was associated with more sleep difficulties [24,25]. Going one step further, school absenteeism, as the strongest expression of school maladjustment and problems, has also been related to sleep dysregulation [26]. Conversely, regarding the positive aspects, school connectedness, which reflects the overall relationship and feelings about school, and school engagement were found to be positively related to sleep quality [27]. At the same time, previous studies underlined that appropriate sleep quality and duration could impact adolescents’ school experience [28], suggesting that better sleep quality may lead to better cognitive and emotional functioning, which can, in turn, be associated with the quality of adolescents’ school experience. However, less attention has been paid to this direction, emphasizing the need to provide a clearer picture of the interplay between sleep quality and school experience over time.

Finally, characteristics of the school structure and environment likely influence sleep. The school ecosystem, including the level of urbanicity, the median household income, and a stressful school environment (both in terms of overload of school homework and activities and a negative relationship with peers), could play a crucial role in several health aspects of adolescents, including sleep. The school starting time is one of the main essential structural factors of the school social context that could disrupt sleep during adolescence [29]. This factor was widely discussed in previous literature, spotlighting the potential advantages of delaying school starting time for increasing sleep duration, regulating sleep schedule, and improving health outcomes [30]. 

Overall, substantial evidence suggests a relationship between adolescents’ school experience and sleep health. However, this was mainly based on cross-sectional studies, leaving it unclear how multiple sleep indicators are developmentally related to adolescents’ school experience. Notably, the prevalent direction of this dynamic relationship and which factor (considering multiple aspects of both school experience and sleep health) has the leading impact on the other remain unclear. For this reason, it is essential to address this gap, comprehensively examining the longitudinal interplay between multiple aspects of sleep and school experience in adolescents.

### 1.3. The Present Study

Healthy sleep in adolescence is a complex construct influenced by individual determinants and the social environment, such as their school experience. Concurrently, unhealthy sleep patterns might influence several factors in the school experience of youths. For this reason, within a socio-ecological approach to healthy sleep development, it is of the utmost importance to disentangle the complex interplay between adolescents’ sleep and their school experience [31]. Therefore, the present systematic review aims to summarize the longitudinal findings on the reciprocal relationship between different aspects of sleep (i.e., sleep duration and sleep schedule, sleep quality, and sleep disturbances) and (a) school performance; (b) the psychological aspects related to the school environment; and (c) the school structure, environment, and schedule. 

## 2. Materials and Methods

This study was conducted following the PRISMA guidelines (Preferred Reporting Items for Systematic Reviews and Meta-Analyses) [32]. The PRISMA checklist is available in the supplemental materials (Document S1). This systematic review was preregistered in the PROSPERO database, registration ID: CRD42021281002. The current study is part of a larger project aiming to review longitudinal research studying the interplay between sleep quality and several proximal (e.g., peers [33], family [34]) and distal (e.g., macro-context [35]) factors in adolescence. 

### 2.1. Eligibility Criteria

Following the PRISMA guidelines [36], specific eligibility criteria were defined [37]. With regards to the study characteristics, studies were eligible for the systematic review if (a) the participants were adolescents from the general population attending junior or secondary high schools (aged between 10 and 19 years old); (b) the study design was longitudinal (with at least two assessments, such as two-wave longitudinal studies or daily diaries); (c) the studies examined at least one aspect of sleep and one of school experience (for an overview of the main dimensions, see Table 1); (d) sleep was measured with either objective (e.g., actigraphy, polysomnography) or subjective standardized measures (e.g., sleep diaries; questionnaires).

Regarding the publication’s characteristics, journal articles and grey literature that can be retrieved through database searches (e.g., doctoral dissertations) were included to avoid selection biases and strengthen the methodological rigor of the systematic review [38]. Finally, no restrictions were applied based on the year and the language of publication (when articles/dissertations were published in a language other than English, professional translators were contacted).

### 2.2. Literature Search 

In order to systematically identify eligible relevant research published in peer-reviewed journal articles or available as grey literature, different search strategies were applied. The literature search was conducted on 23 September 2021, with an update on 28 January 2023. First, several bibliographic databases were systematically searched: Web of Science, Scopus, PsycINFO, PsycArticles, PubMed, MEDLINE, ERIC, ProQuest Dissertations and Theses, and GreyNet. In each database, the following combination of keywords was searched for: (Sleep* OR insomnia* OR polysomnogra* OR REM OR actigraph* OR EEG* OR motor activity* OR circadian* OR chronotype*) AND (pediatr* OR paediatr* OR teen* OR school* OR adolescen* OR youth* OR young* OR child*) AND (longitudinal* OR prospective* OR follow up* OR daily* OR day-to-day OR wave*). Full query strings used in each database are reported in the supplemental materials (Document S2).

This main bibliographic search was complemented with additional search strategies. The websites of the journals deemed most likely to publish studies on the topic were searched, identifying them using the statistics of the previous search on Web of Science, and looking for the fifteen journals in which most articles matching our search strategy had been published (a full list of journals is provided in the supplemental materials, Document S3). This search was performed to identify in-press articles (e.g., online first) which matched the eligibility criteria. Furthermore, conference proceedings from recent sleep-related journals were screened (Journal of Sleep Research, in which the European Sleep Research Society Congress proceedings were published, and Sleep Medicine, in which the World Sleep Congress proceedings were published). The reference lists of the most relevant published systematic reviews and meta-analyses were checked, e.g., [39] (the complete list is reported in the supplemental materials, Document S4). Finally, the reference lists of included studies were checked to further identify relevant studies not initially found through the other search strategies (this search was performed at the end of the selection process). The searches and the screening were run and managed on Citavi 6 software.

### 2.3. Selection of Studies

**Figure 1:**
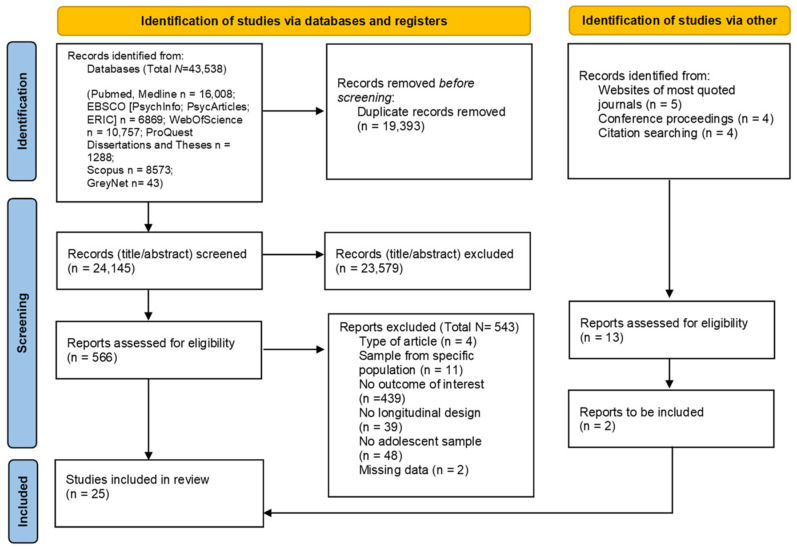
PRISMA diagram search flow.

The results of the search strategies are reported in the PRISMA diagram (Figure 1). A total of 43,538 abstracts were identified, and from these, 19,393 duplicates were removed. Two independent raters screened the remaining records (*N* = 24,145) independently and simultaneously. The percentage of agreement was substantial (Cohen’s Kappa = 0.77). Discrepancies were discussed with a third rater, and the final decisions were taken when the agreement was reached among all three evaluators. 

A total of 566 records were selected at this step. Next, the full texts were screened following the same procedure used for the abstract screening (the agreement was high; Cohen’s Kappa = 0.73). In total, 25 studies were included in this systematic review.

### 2.4. Coding of Primary Studies

In order to extract relevant information from the selected primary studies, an Excel spreadsheet was prepared. All the included studies were coded independently and simultaneously by two independent raters (the percentage of agreement was 96%). Discrepancies were discussed with a third rater and resolved among the three evaluators. 

First, the characteristics of the publication were coded: type of publication (i.e., journal article or grey literature), year of publication, and language of publication. Second, the characteristics of the studies were coded: funding sources (i.e., international, national funding, local funding, multiple funding sources); the number of waves of the longitudinal design; the interval between waves (in months); the dimensions of each study (coded according to the variables presented in Table 1); and the source of information used to evaluate them (i.e., self-reports, objective assessment). Third, the characteristics of the participants were coded: sample size, gender composition of the sample (% females), mean age, geographical location, and ethnic composition of the sample. 

Finally, for each study, a statistical indicator of the strength of the investigated effect was extracted. Specifically, bivariate correlations, regression, odd ratio coefficients, mean and standard deviation, and Cohen’s *d* were used to estimate the interplay between sleep variables and (a) school performance; (b) the psychological aspects related to the school environment; and (c) the school structure, environment, and schedule. When available, the cross-lagged bivariate associations were based on the specific school experience dimension measured at one time point (e.g., school engagement at T1) and sleep dimension at a later time point (at T2), or sleep dimension at one time point (e.g., objective sleep quality at T1) and the school experience dimension variable measured at a later time point (e.g., grades at T2). Qualitative information on the main findings were extracted when primary studies did not report effect sizes.

### 2.5. Methodological Quality Assessment and Risk of Bias 

In line with the PRISMA guidelines [36], each study’s methodology quality and risk of bias were evaluated. The first author performed the quality assessment of studies by using an adapted version of the Newcastle–Ottawa Scale (NOS) for cohort studies [40]. Additionally, the second author independently evaluated a subsample of references (40% of the total, with a high percentage of agreement: 80%). Discrepancies were discussed with a third rater and resolved among the three evaluators. Since the current systematic review only included longitudinal studies, the assessment areas of the scale were adapted to the relevant characteristics of the specific study design, as in previous research (e.g., [35]). Specifically, the adapted version of the NOS included six items categorized into three dimensions: Selection, Comparability, and Outcome. A series of response options are provided for each item. A star system is used, assigning a maximum of one star for each Selection and Outcome item and a maximum of two stars for the Comparability item to high-quality studies. The full list of items is available in Document S5 of the Supplemental Materials.

## 3. Results

### 3.1. Study Characteristics

Twenty-five studies were included in the systematic review. A summary of the characteristics of the included studies is reported in Table 2. Regarding the characteristics of the publication, all the studies were articles published in peer-reviewed journals and in the English language. In terms of the year of publication, most of them (60%) were published recently, between 2018 and 2023, while the rest were published between 2011 and 2017 (24%) or before 2008 (16%). With regards to the study design, one study used a daily study design; around half of the studies (56%) included two time points; and the remaining studies included three time points (24%) or more than three time points (16%). The average time lag between adjacent waves was approximately one year (*M* = 12.6 months; *SD* = 8.6 months), ranging from 1 month to 3 years. Only three studies [41,42,43] assessed sleep duration using an objective measure (i.e., actigraphy), and the remaining studies used self-report measures. Most studies (72%) reported one or multiple funding sources. The total number of participants was 47,756 (*M* = 1910.2, *SD* = 2256.2, range 33–7394). Most samples were gender-balanced (the average percentage of females across samples was 52.1%; range 39.5%–65.3%), and the average age of the sample participants at baseline was 14.9 years (*SD* = 14.1, range: 12–16.9 years). With regards to the geographic context of the studies, most were conducted in the USA (36%), Europe (20%), China (20%), and Japan (8%); the remaining samples were from Australia [42], Brazil [44]; Canada [45], and South Korea [46].

### 3.2. Methodological Quality and Risk of Bias of the Studies

The results of the methodological quality and risk of bias assessment are reported in Document S6 of the Supplemental Materials. Specifically, 16 of the 25 included studies displayed high quality, while the remaining showed medium quality. Thus, the overall quality of the studies was high, with a consequent low risk of bias.

### 3.3. Associations between Sleep and School Experience

This systematic review highlighted that the selected primary studies examined a heterogeneous array of relationships between sleep and school context dimensions. In accordance with the main aims of this work, the results were organized into three main clusters: (a) sleep and school performance, (b) sleep and psychological aspects of school, and (c) sleep and school structure and environment. For each cluster, the main results are examined, focusing on the direction of the effects (from sleep dimension at one time point to school experience dimension at a later time point, or from school experience at one time point to sleep dimension at a later time point), the time lag (e.g., effects in the short, medium, or long term), and the assessment method.

#### 3.3.1. Longitudinal Associations between Sleep and School Performance

Seven studies evaluated the interconnection between sleep (i.e., sleep duration and sleep schedule, sleep disturbances) and school performance (i.e., grades). The main findings are summarized in Table 3. Of these studies, three [24,51,57] evaluated the bidirectional long-term effect between subjective sleep disturbances, and duration and grades. The results highlighted a negative bidirectional relationship between sleep disturbances and grades [24] and a positive relationship between longer sleep duration and grades over time [51,57], with slight effects in the opposite direction highlighted by only one study [51]. Two studies [47,61] focused on the specific long-term effect of subjective sleep schedule, duration, and sleep disturbances on grades. The results showed that the presence of a late bedtime and sleep disturbances, but not sleep duration, was predictive of lower cumulative grades over time. Two studies [50,60] did not find any short- or long-term effect in assessing the influence of subjective sleep duration on grades.

#### 3.3.2. Longitudinal Associations between Sleep and Psychological Aspects of Educational Context

Eight studies evaluated the interconnection between sleep indicators (i.e., sleep quality and sleep disturbances) and the psychological aspects of the school experience (i.e., school burnout, school connectedness, engagement, absenteeism, avoidance, school problems, and school bullying). The main findings are summarized in Table 4. Specifically, four studies evaluated the bidirectional long-term effect between subjective sleep quality and disturbances and the psychological aspects of the educational context [24,25,27,53]. The results of these studies underlined that poor sleep quality and disturbances were bidirectionally associated with higher levels of school burnout, lower levels of school connectedness, and higher levels of school bullying. Four studies tackled how sleep contributed to the educational experience’s psychological aspects. On the one hand, three studies showed that higher levels of self-reported sleep disturbances were correlated with increased absenteeism and school-related problems in the long term [48,54], with one study highlighting this association only in females [52]; on the other hand, one study [50] showed that subjective sleep quality, but not sleep duration, was strongly associated with higher school engagement over a long-term period.

#### 3.3.3. Longitudinal Association between Sleep and School Structure and Environment

Thirteen studies evaluated the relationship between sleep variables (i.e., sleep duration and sleep schedule; sleep quality) and school structure and environment (i.e., school activities; stressful school environment; school schedule). The main findings are summarized in Table 5.

- **School Activities.**

Two studies [46,55] evaluated the impact of the amount of time spent on school activities and school work on later levels of subjective nocturnal sleep duration. The results were mixed; while one study showed a long-term effect of being involved in school activities in shortening sleep duration [55], the other study did not find the same relationship [46]. Conversely, another study [56] evaluated the opposite direction, highlighting that self-reported sleep disturbances pose a significant risk for involvement in school activities and school homework difficulties.

- **School Stressful Environment.**

Regarding the school environment, two studies assessed the unidirectional impact of a disadvantaged school context and peer victimization exposure on subjective sleep duration and quality in the long term [45,59]. The results showed that these two stressful characteristics of the school environment were related to shorter sleep durations and worse sleep quality.

- **School Schedule.**

Notably, the results of three (out of four) studies [29,47,49,58] showed a unidirectional effect of a school schedule with a later school starting time in delaying bedtime and waketime and increasing sleep duration, both in the short and long term. Conversely, four (out of five) studies [29,41,42,43,44] underlined the unidirectional effects of the early school start time in decreasing nocturnal sleep duration and delaying bedtime (assessed with both objective and subjective measures), but not an impact on waketime and sleep quality both in the short and long term. 

## 4. Discussion

Sleep is a critical psychophysiological process influencing the health and development of adolescents [15,62]. Previous research has shown that poor sleep quality is crucial in negatively affecting youth learning capacity, emotional stability, and neurobehavioral functioning [10]. At the same time, adolescence is a period that particularly challenges healthy sleep habits due to developmental changes in cognitive, behavioral, and emotional functioning, and new roles and demands in the familiar and social context. Therefore, healthy sleep during adolescence is likely to be bidirectionally linked with multiple developmental and environmental systems [63]. One of the main factors that might affect sleep quality and its development in adolescents is the school experience [7].

Similar to what happens in adulthood, when the job context has a crucial impact on different aspects of health and vice versa, adolescents spend a large proportion of their waking hours performing activities related to the school experience, which could, in turn, be affected by poor sleep quality. Although much of the literature highlighted an association between specific sleep variables and school experiences, most was based on cross-sectional research. Moving a step further, this systematic review aimed to understand the longitudinal and bidirectional relationship between healthy sleep patterns and adolescents’ school experience. The main results highlighted that: (a) unhealthy sleep patterns are interconnected with poor school performance; (b) poor sleep quality and sleep disturbances are bidirectionally related to both negative (i.e., higher school burnout and problems) and positive (i.e., lower levels of school connectedness) psychological aspects related to the school experience; and (c) school structural characteristics affect the sleep duration and schedule of adolescents over time. Below, we discuss these main findings, considering their implications for future research and clinical practice.

### 4.1. Interconnection between Unhealthy Sleep and Poor School Performance

School performance and, specifically, adolescents’ grades are affected by several psychosocial factors, such as intelligence, socio-economic status, motivation, and health problems [64]. This review also documented a longitudinal association between unhealthy sleep (i.e., short sleep duration, difficulties in initiating and maintaining sleep) and later poor school performance [47]. In addition, a few studies [24,51,59] evaluated the bidirectional relationship between sleep duration, schedule, and grades over time. The results showed that sleep disturbances and short sleep duration predict school performance, highlighting a negative association between sleep difficulties and grades over time. At the same time, these studies underlined the role of poor school performance in predicting higher sleep disturbances and shorter sleep duration over time. The longitudinal impact of sleep disturbances on adolescents’ school success can be explained through several mechanisms, such as increased daytime sleepiness and decreased motivation for general health due to sleep debt [65]. Furthermore, this could establish a vicious circle in which worse school performance could have negative consequences at a psychological level (e.g., decreasing self-efficacy and increasing anxiety), worsening sleep health in turn.

The results of this systematic review confirm previous cross-sectional research that evaluated only specific aspects of sleep without considering the whole physiological facets of sleep, reporting mixed results and a stronger association between poor sleep quality and lower grades than sleep duration [66]. Future studies should comprehensively investigate all aspects of sleep, using both objective and subjective measurements, and how these factors interact with grades over time. It would thus be possible to recognize which factors of healthy sleep behaviors have a stronger impact on this vital aspect of adolescents’ lives and develop appropriate interventions and educational care policies.

### 4.2. Unhealthy Sleep Patterns and the Reciprocal Association with Positive and Negative Psychological Factors at School 

Poor sleep quality in adolescents can affect many psychosocial functions [2]. This review contributes to the literature by highlighting the bidirectional relationship between poor sleep quality, sleep disturbances, and school-related psychological factors. More specifically, poor sleep quality and sleep disturbances were associated with higher levels of school burnout, lower levels of school connectedness, and higher levels of school bullying and vice versa [24,25,27,53]. This evidence points to potential vicious circles in which stressful environments could exacerbate mechanisms representing risk factors for sleep problems, such as rumination and high arousal levels [67]. Consequently, sleep difficulties, in turn, are likely to affect the school experience at a psychological level, increasing school burnout and decreasing school connectedness.

Furthermore, considering studies that evaluated the unidirectional effect of sleep on psychological factors related to the school experience, the results showed that higher sleep quality and disturbances, but not sleep duration, were associated with a higher level of positive psychological factors (i.e., school engagement) and a lower level of negative psychological factors (i.e., school problems) over time [48,50,54]. This systematic review pointed out the importance of considering the sleep dimension in the school experience for adolescents’ psychological health and development. Understandably, fostering a positive and healthy school experience climate is essential to promoting the psychological well-being of youth. At the same time, restorative sleep is crucial for a positive school experience (e.g., increasing school engagement and decreasing absenteeism).

### 4.3. School Structure and Environment Influence Sleep Duration and Schedule 

The current systematic review highlighted how school structure and environment characteristics could significantly impact sleep variables over time. The included studies mostly investigated the unidirectional effect of school structural aspects on adolescents’ sleep health. The results showed that stressful school environments (i.e., exposure to peer victimization, stressful school environment, and disadvantaged school area) were connected to shorter sleep duration and worse sleep quality over time [45,59]. Only one study [56] evaluated the unidirectional effect of sleep disturbances on school activities and homework involvement. The results showed that sleep disturbances were negatively associated with adolescents’ school activities and homework involvement. 

Furthermore, considerable attention was paid to the role played by the school schedule. The results showed that late school start time had a longitudinal impact on increasing sleep duration and delaying sleep schedule, and that early school start time had a longitudinal effect on decreasing sleep duration and delaying sleep schedule [28,48,49,58]. These findings are in line with the Perfect Storm model [8] that highlights the crucial role of the school start time, forcing adolescents to be awake earlier than spontaneous arousal using an alarm clock, which is incompatible with several aspects of modern society [7]. Several psychological and physical adverse health outcomes are associated with sleep debt, including mood disturbances, behavioral problems, weight gain, and health issues (for a review, see [10,12,68]). Notably, delaying school start times could have positive implications for adolescents’ sleep health and beneficial societal effects in decreasing motor vehicle accident risk, increasing community education regarding adolescent sleep need, and providing significant economic gains due to higher school performance [69,70]. Nevertheless, longitudinal studies reporting the influence of school-related aspects on sleep quality and quantity would be necessary for public health in order to avoid one-size-fits-all benchmarks that can be difficult and unnecessary for some youth to achieve.

### 4.4. Limitations and Suggestions for Future Research 

The studies included in this systematic review were heterogeneous in some crucial variables. For this reason, a quantitative meta-analytic summary would not be informative in this case. This suggests the need for future longitudinal studies evaluating different aspects of sleep in relation to school experience, with standardized and comparable measures that could facilitate the possibility of conducting a meta-analysis and obtaining an overall estimate of this association. In addition, most of the included studies used self-reported measures susceptible to self-perception bias. Future studies should integrate these findings with variables collected using both objective (i.e., actigraphy and polysomnography) and subjective (i.e., standardized questionnaires and sleep diaries) measures to capture the most accurate picture of the phenomenon. Since actigraphy is an unobtrusive instrument that uses validated algorithms and standardized scoring procedures, it is of the utmost importance to employ it in longitudinal designs assessing adolescents’ healthy sleep patterns [71]. Furthermore, this systematic review focused specifically on community samples of adolescents. Therefore, the results of this review cannot be generalized to clinical populations. Future studies should also replicate these findings in clinical samples in order to implement specific guidelines for the healthy development of adolescents in relation to their school experience. Finally, this systematic review included studies in which specific aspects of the school experience were considered separately in relation to adolescents’ sleep. However, adolescents live within a complex and dynamic system that significantly influences their sleep health. For this reason, in line with the ecological model [72], it is of the utmost importance to evaluate how school experience interacts with other crucial social systems of adolescents (e.g., familiar context, relational difficulties) and, consequently, how it impacts adolescents’ health.

## 5. Conclusions

This systematic review provided a comprehensive synthesis of longitudinal research on the relationships between sleep and adolescent school experience. First, the results highlighted that unhealthy sleep and sleep disturbances, but not sleep duration, predict school performance over time. Second, the findings showed a bidirectional longitudinal relationship between sleep disturbances, poor sleep quality, and psychological aspects (i.e., school engagement, burnout, and connectedness) during adolescence. These results suggest that the psychological factors related to the school experience that are expected to affect healthy sleep later are likely to be affected by sleep itself. Finally, school structure and environment seem to have a longitudinal impact on adolescents’ sleep duration and sleep schedule. 

If, on the one hand, this review provides novel insights on the bidirectional relationship between school experience and sleep health, on the other, it calls for more longitudinal research investigating all aspects of healthy sleep, and the size and direction of the association. In fact, despite the recent efforts of the world’s scientific community to improve adolescents’ sleep, future longitudinal studies must be conducted using both objective and subjective measures (e.g., the combination of both standardized questionnaires and actigraphy) to better understand the direction of the association between sleep and psychological factors and target specific aspects of both sleep and school domains that could be enhanced. Thus, specific early preventive and promotional interventions could be implemented to address the identified variables, which might prove fundamental for strengthening healthy sleep and the overall school experience.

## Figures and Tables

**Table 1:** Main categories of sleep and school experience dimensions.

**Sleep**		
**Sleep Quantity**	**Sleep Quality**	**Sleep Disturbances**
Sleep duration	Sleep efficiency	Difficulties in falling asleep
Sleep schedule (bedtime and waketime)	Sleep onset latency	Difficulties in sleep maintenance
	Nocturnal wakefulness	
	Perceived sleep quality	
	**School Experience**	
**Academic Performance**	**Psychological Aspects of the School Experience**	**School Structure and Environment**
Grades	School burnout	School area
	Absenteeism	Stressful school environment
	School problems	School activities and homework
	School engagement and connectedness	School starting time schedule

**Table 2:** Characteristics of Studies.

Study	Characteristics of the Studies							Characteristics of the Participants				
Authors and year	Funding	Number of waves	Interval between waves (months)	Sleep variable	Sleep assessment	School-related variable	School-related assessment	Country	% Female	Mean age	Sample size	White/European; Black/African American; Asian; Hispanic (%)
Alfonsi et al., 2020 [29]	n/a	6	1	Sleep duration and sleep schedule	Subjective	School structure and environment	Self-report	Italy	52.9	14.1	51	n/a
Andrade et al., 1993 [44]	Local funding	3	6	Sleep duration and sleep schedule	Subjective	School structure and environment	Self-report	Brazil	48.4	13.6	66	n/a
Asarnow et al., 2014 [47]	Multiple funding	3	12–60	Sleep duration and sleep schedule	Subjective	School performance; school schedule	Institution	USA	55	14.8	2700	61.6; 22.0; 4.2; 7.7
Bao et al., 2018 [27]	n/a	2	9	Sleep quality	Subjective	Psychological aspects of school	Self-report	China	54.8	14.9	888	n/a
Bauduccco et al., 2015 [48]	n/a	2	12	Sleep disturbances	Subjective	Psychological aspects of school	Self-report	Sweden	48.1	n/a	353	n/a
Biller et al., 2021 [49]	No	2	12	Sleep duration and sleep schedule	Subjective	School structure and environment	Self-report	Germany	60	15.8	33	n/a
Dunbar et al., 2017 [50]	No	6	6	Sleep duration and sleep schedule	Subjective	School performance; school engagement	Institution	USA	64.1	14.4	310	22.7; 10.7; 38.0; 24.0
Evers et al., 2020 [24]	Multiple funding	2	5	Sleep disturbances	Subjective	School performance; school burnout	Self-report	China	47.5	13.9	2283	n/a
Fredriksen et al., 2004 [51]	Local funding	3	12	Sleep duration and sleep schedule	Subjective	School performance	Self-report	USA	49.6	n/a	2259	82.2; 5.4; 3.4; 6.9
Liu et al., 2021 [25]	Multiple funding	4	6	Sleep disturbances	Subjective	Psychological aspects of school	Self-report	China	50.3	12.7	1226	n/a
Fujimura et al., 2023 [52]	Multiple funding	2	36	Sleep quality	Subjective	Psychological aspects of school	Self-report	Japan	50.1	12.2	6077	n/a
He et al., 2022 [53]	National funding	2	12	Sleep quality	Subjective	Psychological aspects of school	Self-report	China	39.5	12.5	1687	n/a
Mitchell et al., 2020 [41]	Multiple funding	2	12	Sleep duration and sleep schedule	Objective	School structure and environment	Self-report	USA	50	13.9	84	69.0; 25.2; n/a; n/a
Patte et al., 2017 [45]	National funding	3	12	Sleep duration and sleep schedule	Subjective	School structure and environment	Self-report	Canada	53.9	n/a	7394	70.8; 3.3; 4.8; 1.5
Roberts et al., 2002 [54]	National funding	2	12	Sleep disturbances	Subjective	Psychological aspects of school	Self-report	USA	49.2	n/a	3136	37.0; 34.6; n/a; 23.7
Roberts et al., 2008 [55]	National funding	2	12	Sleep disturbances	Subjective	School structure and environment	Self-report	USA	49.2	n/a	3134	37.0; 34.6; n/a; 23.7
Roberts et al., 2011 [56]	National funding	2	12	Sleep disturbances	Subjective	School structure and environment	Self-report	USA	49.2	n/a	3134	37.0; 34.6; n/a; 23.7
Shen et al., 2021 [42]	National funding	Daily	Daily	Sleep duration and sleep schedule	Objective and subjective	School structure and environment	Self-report	Australia	54.1	16.9	204	35.6; n/a; 55.6; n/a
Stefansdottir et al., 2020 [43]	National funding	2	24	Sleep duration and sleep schedule	Objective and subjective	School structure and environment	Self-report	Iceland	65.3	n/a	145	n/a
Takizawa & Kobayashi, 2022 [57]	No	2	36	Sleep duration	Subjective	School performance	Self-report	Japan	58.3	n/a	139	n/a
Thacher et al., 2016 [58]	n/a	3	6	Sleep duration and sleep schedule	Subjective	School structure and environment	Self-report	USA	n/a	16.7	372	n/a
Tu et al., 2019 [59]	National funding	2	10	Sleep quality	Subjective	School structure and environment	Self-report	USA	50	12	123	58.5; 35.0; n/a; n/a
Yoo, 2020 [46]	Multiple fundings	4	12	Sleep duration and sleep schedule	Subjective	School structure and environment	Self-report	South Korea	47	n/a	4335	n/a
Vedøy et al., 2021 [60]	National funding	3	12	Sleep duration and sleep schedule	Subjective	School performance	Institution	Norway	54.4	13.3	552	n/a
Zhang et al., 2022 [61]	National funding	2	12	Sleep disturbances	Subjective	School performance	Self-report	China	50	14.6	7072	n/a

**Table 3:** Longitudinal associations between sleep and school performance.

Study	Sleep Variables	School Experience Variable	Main Findings	Sleep T1 → School Experience T2	School Experience T1 → Sleep T2
Asarnow et al., 2014 [47]	Late bedtime	Grades	Late bedtime at one time point was predictive of lower cumulative grades at a later time point.	*β* = −0.27 *** [−0.37, 0.17]	
	Short sleep duration		Short sleep duration at one time point was not predictive of grades at a later time point.	*β* = 0.07 [−0.02, 0.15]	
Dunbar et al., 2017 [50]	Sleep duration	Grades	No effect was found between sleep duration and grades over time.	*B* = −0.11 (0.07)	
Evers et al., 2020 [24]	Sleep disturbances	Grades	Sleep disturbances at one time point were significantly and negatively correlated to school performance at a later time point and vice versa.	*r* = −0.19 ***	*r* = −0.16 ***
Fredriksen et al., 2004 [51]	Sleep duration	Grades	Longer sleep duration at one time point was positively and significantly correlated with higher self-reported grades at a later time point. The correlation in the opposite direction was also found but slighter.	*r* = 0.16 *	*r* = 0.08 ***
Takizawa & Kobayashi, 2022 [57]	Sleep duration	Grades	Longer sleep duration at one time point was positively and significantly correlated with higher self-reported grades in math and Japanese at a later time point. No effect was found for correlation in the opposite direction.	Japanese grades: *r* = 0.24 ** Math grades: *r* = 0.26 **	Japanese grades: *r* = 0.07 Math grades: *r =* 0.07
Vedøy et al., 2021 [60]	Sleep duration	Grades	No effect was found between sleep duration and grades over time.	*β* = −0.24 [0.03, 0.46]	
Zhang et al., 2022 [61]	Sleep disturbances	Grades	Sleep disturbances at one time point were significantly and negatively correlated to school performance at a later time point.	OR = 1.30 * [1.06–1.59]	

Note. Sleep T1 → School experience T2 = relationship between the sleep variable measured at one time (T1) point and school experience variable measured at a following time point (T2). School experience T1 → sleep variable T2 = relationship between the school experience measured at one time (T1) point and the sleep variable measured at the following time point (T2). *r* = correlation coefficient; *B* = unstandardized regression coefficient and standard error estimate in parenthesis; *β* = standardized regression coefficient and confidence interval in square brackets; OR [CI]: odd ratio with confidence interval in square brackets. *** *p* < 0.001, ** *p* < 0.01, * *p* < 0.05.

**Table 4:** Longitudinal associations between sleep and psychological aspects of school.

Study	Sleep Variables	School Experience Variable	Main Findings	Sleep T1 → School Experience T2	School Experience T1 → Sleep T2
Bao et al., 2018 [27]	Poor sleep quality	School connectedness	A higher level of school connectedness at one time point was negatively and significantly correlated with fewer sleep quality problems at a later time point and vice versa.	*r* = −0.19 ***	*r* = −0.18 ***
Bauducco et al., 2015 [48]	Sleep disturbances	Absenteeism	A higher level of absenteeism at one time point was positively and significantly correlated with more severe sleep problems at a later time point.	*B* = 1.07 * (0.44)	
Dunbar et al., 2017 [50]	Sleep quality	School engagement	Sleep quality at one-time point was predictive of higher school engagement at a later time point.	*B* = 0.26 *** (0.05)	
	Sleep duration		No effect was found between sleep duration and school engagement over time.	*B* = −0.05 (0.03)	
Evers et al., 2020 [24]	Sleep disturbances	School burnout	Sleep disturbances at one time point were significantly and positively correlated to burnout at a later time point and vice versa.	*r* = 0.20 ***	*r* = 0.22 ***
Fujimura et al., 2023 [52]	Sleep onset latency	School avoidance	School avoidance at one time point poses a significant risk for long sleep onset latency at a later time point in females but not in males.		Males: OR: 1.24 [0.91–1.69] Females: 1.55 ** [1.16–2.08]
He et al., 2022 [53]	Poor sleep quality	School bullying	Poor sleep quality at one time point was significantly and positively correlated to school bullying at a later time point and vice versa.	*r* = 0.16 **	*r* = 0.11 **
Liu et al., 2021 [25]	Poor sleep quality	School burnout	Poor sleep quality at one time point was significantly and positively correlated to burnout at a later time point and vice versa.	*r* = 0.25 ***	*r* = 0.43 ***
Roberts et al., 2002 [54]	Sleep disturbances	School problems	Sleep disturbances at one time point pose a significant risk for school problems at a later time point, even after controlling for baseline functioning, age, gender, and parental education.	OR: 2.65 *** [1.99, 3.52]	

Note. Sleep T1 → School experience T2 = relationship between the sleep variable measured at one time (T1) point and school experience variable measured at a following time point (T2). School experience T1 → sleep variable T2 = relationship between the school experience measured at one time (T1) point and the sleep variable measured at the following time point (T2). *R* = correlation coefficient; OR [CI]: odds ratio coefficient and confidence interval in square brackets, *B* = unstandardized regression coefficient and standard error estimate in parenthesis; *** *p* < 0.001, ** *p* < 0.01, * *p* < 0.05.

**Table 5:** Longitudinal association between sleep, and school structure and environment.

Study	Sleep Variables	School Experience Variable	Main Findings	Sleep T1 → School Experience T2	School Experience T1 → Sleep T2
Alfonsi et al., 2020 [29]	Sleep duration	Early school start time	Early school start time at one time point was associated with decreased sleep duration at a later time point.		−0.01% ± 0.03 (−11.40 ± 13.30 min of sleep)
	Sleep duration	Late school start time	Late school start time at one time point was associated with increased sleep duration at a later time point.		+ 8.90% ± 0.03 (+34.10 ± 11.90 min of sleep)
Andrade et al., 1993 [44]	Bedtime	Early school start time	No effect of the early school start time was found on sleep schedule and duration over time.		
	Waketime				
	Sleep duration				
Asarnow et al., 2014 [47]	Bedtime	Late school start time	Late school start time at one time point was associated with a delay in bedtime at a later time point.		+1% > 11:15 P.M.
	Sleep duration		Late school start time at one time point was associated with increased sleep duration at a later time point.		+7% > 9 h
Biller et al., 2021 [49]	Sleep duration	Late school start time	No effect found.		F(2,31) = 0.54 η2 = 0.03, *d* = 0.37
	Bedtime		No effect found.		F(2,31) = 1.61 η2 = 0.09, *d* = 0.64
	Waketime		Late school start time at one time point was associated with a delay of waketime at a later time point.		F(2,31) = 9.03 *** η2 = 0.36, *d* = 1.52
Mitchell et al., 2020 [41]	Sleep duration	Early school start time	Early school start time at one time point was associated with decreased sleep duration at a later time point.		<25.8 min
	Bedtime		Early school start time at one time point was associated with a delay in bedtime at a later time point.		>22.2 min
Patte et al., 2017 [45]	Sleep duration	Disadvantaged school area	A disadvantaged school’s geographical area increased the likelihood of short sleep duration over time.		>probability of reporting short sleep duration if attended schools in large urban areas and where the median annual household income was USD 50,000–75,000
Roberts et al., 2008 [55]	Sleep disturbances	School activities	Sleep disturbances at one time point pose a significant risk for involvement in school activities and difficulties at a later time point.	OR: 3.23 * [1.78–5.88]	
		School homework	Sleep disturbances at one time point pose a significant risk for involvement in school homework difficulties at a later time point.	OR: 2.86 * [1.70–4.81]	
Roberts et al., 2011 [56]	Short sleep duration	School activities	Schoolwork at one time point poses a significant risk for short sleep duration at a later time point.		OR: 1.60 * [1.03–2.78]
		School homework	School activities at one time point pose a significant risk for short sleep duration at a later time point.		OR: 0.89 * [0.49–1.60]
Shen et al., 2021 [42]	Sleep duration	Early school start time	Early school start time at one time point was associated with decreased sleep duration at a later time point.		<total sleep time (7.8 ± 1.03 vs. 8.01 ± 1.07); >sleep debt (0.33 ±1.1 vs. −0.1 ±1.3) ***
Stefansdottir et al., 2020 [43]	Sleep duration	Early school start time	Early school start time at one time point was associated with decreased sleep duration at a later time point.		T1: 7.2 ± 0.7; T2: 6.7 ± 0.8 ***
	Sleep efficiency		No effect found.		T1:88.7 ± 3.8; T2: 88.6 ± 4.3
Thacher et al., 2016 [58]	Sleep quality	Late school start time	Late school start time at one time point was associated with increased sleep duration at a later time point.		T1: 5.5 ± 0.1; 6.03 ± 0.1
	Bedtime		Late school start time at one time point was associated with a delay in bedtime at a later time point.		T1: 22:35 ± 74 min; T2: 22:58 ± 76 min
	Waketime		Late school start time at one time point was associated with a delay of waketime at a later time point.		T1: 6:55 ± 50 min; T2: 7:02 ± 50 min
Tu et al., 2019 [59]	Sleep quality	Stressful school environment	Peer victimization exposure at school at one time point was significantly and negatively correlated to sleep quality at a later time point.		*r* = −0.46 ***
Yoo, 2020 [46]	Sleep duration	School activities	No effect found.		*B* = 0.04 (0.04)

Note. Sleep T1 → School experience T2 = relationship between the sleep variable measured at one time (T1) point and school experience variable measured at a following time point (T2). School experience T1 → sleep variable T2 = relationship between the school experience measured at one time (T1) point and the sleep variable measured at a following time point (T2). OR [CI]: odds ratio coefficient and confidence interval in square brackets; *r* = correlation coefficient; η2, (partial) eta squared; *d* = Cohen’s d coefficient, *B* = unstandardized regression coefficient and standard error estimate in parenthesis; *** *p* < 0.001, * *p* < 0.05.

## Data Availability

Data from previously published studies were retrieved and analyzed. Data sharing is not applicable to this article.

## Supplementary Materials

The following supporting information can be downloaded at: https://www.mdpi.com/article/10.3390/ijerph20064829/s1, Document S1: Prisma checklist; Document S2: Full query string for each database; Document S3: Screened journals; Document S4: Full list of most relevant published systematic reviews and meta-analyses; Document S5: Quality and Risk of Bias Assessment Method (Adapted from the Newcastle–Ottawa Scale for Cohort Studies); Document S6: Risk of Bias Assessment Results. References [39,73,74,75,76,77,78,79,80,81,82,83,84] are cited in the supplementary materials.
